# Gestational hemostasis: a natural model for hemostasis resuscitation of major periprocedural blood loss: *“Look deep into nature, and then you will understand everything better.” Albert Einstein*

**DOI:** 10.1186/s13741-021-00225-0

**Published:** 2021-12-13

**Authors:** Barna Babik, Szilvia Kupcsulik, János Fazakas

**Affiliations:** 1 Department of Anesthesiology and Intensive Therapy, Faculty of Medicine, University of Szeged, Szeged, Hungary; 2 Clinic of Transplantation and Surgery, Semmelweis University, Baross Str. 23, Budapest, Hungary

**Keywords:** Coagulation, Pregnancy, Physiology, Massive bleeding, Massive transfusion

## Abstract

Early goal-directed treatment is an evidence-based approach to guide hemostatic therapy during major periprocedural bleeding. If viscoelastic coagulation tests are not available, an algorithm, termed the pyramid of hemostatic interventions, can help manage severe bleeding. Pregnant women accumulate huge reserves of prothrombotic and antifibrinolytic hemostatic elements to avoid peripartum blood loss. We provide comparison of therapeutic hemostatic approaches and natural gestational process and identified remarkable analogy between early goal-directed management of bleeding and hemostatic adaptation of pregnant woman. Therefore, gestational hemostasis serves as a natural model for goal-directed hemostasis resuscitation and can foster understanding of hemostatic management of periprocedural bleeding.

## Background and aims

Patients with major perioperative bleeding can be managed by the pyramid of hemostatic intervention (Gorlinger 2006) as suggested by earlier publications or more recently by guidelines, consensus statements, or reviews based on point-of-care (POC) tests and algorithms (Kozek-Langenecker 2014; Spahn et al. 2019; Maegele et al. 2017; Rossaint et al. 2016; Schochl et al. 2012).

Adaptive physiological changes during pregnancy include alterations in the hemostatic system (Carlin and Alfirevic 2008). The pregnant woman accumulates notable prothrombotic reserve during the third trimester (Bremme 2003; Cerneca et al. 1997; Faught et al. 1995; Greer 1994; Isermann et al. 2003; Lefkowitz et al. 1996; Stirling et al. 1984; Kruithof et al. 1987; Thornton and Douglas 2010; Hellgren 2003; Uchikova and Ledjev 2005; Valera et al. 2010). The new hemostatic balance fulfills the simultaneous physiological need for blood flow during pregnancy and minimal blood loss at delivery.

The therapeutic interventions during massive bleeding and the natural hemostatic adaptive processes during pregnancy share common goals and physiological mechanisms. We sought to examine the analogies between the early goal-directed clinical management of severe bleeding and the physiological hemostasis. We also aimed at analyzing whether gestational hemostasis may serve as a natural model for early goal-directed individualized hemostasis resuscitation with or without viscoelastic POC tests. Overviewing the hemostasis model of pregnancy may inform the management of perioperative bleeding and may improve patient safety and blood saving.

## Diagnostic and therapeutic steps in managing major perioperative bleeding: the pyramid of hemostatic interventions

**Fig. 1:**
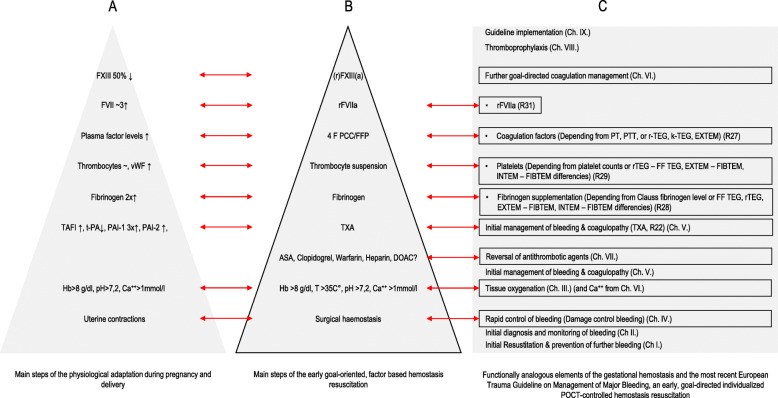
Comparison of global gestational hemostasis (**A**) with the pyramid of interventions (**B**) and the relevant steps of the algorithms reflecting the early, goal-directed, individualized, point-of-care-controlled hemostasis resuscitation (**C**). The analogy between the gestational adaptation as a natural prothrombotic mechanism and the therapeutic algorithms is remarkable (red arrows). (Abbreviations: ASA: aspirin, Ch: chapter, DOAC: direct acting oral anticoagulants, EXTEM: extrinsically triggered rotational thromboelastometry, FF-TEG functional fibrinogen TEG, FIBTEM: cytochalasin D added EXTEM test, FFP: fresh frozen plasma, FVII: coagulation factor VII, FXIII: coagulation factor XIII, Hb: hemoglobin, INTEM: intrinsically triggered rotational thromboelastometry, k-TEG: kaolin TEG, PAI-1: plasminogen activator inhibitor 1, PAI-2: plasminogen activator inhibitor 2, PT: prothrombin time, PTT: partial prothrombin time, rFVIIa: recombinant activated factor VII, (r)FXIII(a): recombinant activated factor XIII, r-TEG: rapid TEG, TAFI: thrombin-activatable-fibrinolysis-inhibitor, TEG: thromboelastography, t-PA: tissue plasminogen activator, TXA: tranexamic acid, vWF: von-Willebrandt factor, 4F PCC: four-factor prothrombin complex concentrate)

In 2006, a simple algorithm was published for managing bleeding during liver transplantation (Gorlinger 2006) (Fig. 1B). This algorithm—often called pyramid or triangle of hemostatic interventions—includes the diagnostic and therapeutic, with the physiological and clinical, aspects required to manage severe perioperative bleeding (Kozek-Langenecker 2014; Rossaint et al. 2016; Babik et al. 2013). This contributes to the later-developed concept of early, goal-directed individualized hemostasis resuscitation controlled by POC hemostatic tests during periprocedural bleeding.

The base of the pyramid focuses on the role of appropriate surgical hemostasis. The importance of an intuitive and accurate surgical approach cannot be overestimated (Rossaint et al. 2016).

The next step in the pyramid involves securing the “optimal tissue environment” of hemostasis (De Robertis et al. 2015). Red blood cells (RBC) propel thrombocytes toward the endothelium and facilitate their aggregation by releasing adenosine-diphosphate (ADP). Maintenance of body core temperature > 35 °C is needed for optimal enzymatic mechanisms, with prevention and treatment of acidosis (pH > 7.2). Serum Ca^2+^ levels must be kept above 1 mmol/l, because Ca^2+^ is important in activation of factors VII, −II, and −X (Rossaint et al. 2016; Babik et al. 2013; De Robertis et al. 2015).

The next step is to assess whether the patient has been previously medicated by any antithrombotic drug. If yes, a specific treatment shall be initiated (Rossaint et al. 2016; Babik et al. 2013).

Fibrinolysis must be assumed periprocedurally as a consequence of large tissue damage, long surgical interventions, or splanchnic hypoperfusion. Tranexamic acid (TXA) can form a circulating reversible complex with plasminogen, preventing it from binding to the clot. The plasma half-life of TXA is 1.5 h; therefore, a loading dose should be followed by infusion (Rossaint et al. 2016). If the infusion is stopped at the end of surgery, bleeding may restart after 1–2 h, often caused by lysis (Rossaint et al. 2016; Babik et al. 2013). In other words, the patient’s clinical and pathophysiological states at the end of surgery are disparate, especially from a hemostatic point of view.

The next important step is the supplementation of fibrinogen, which is the object of consequent prothrombotic enzyme processes, the subject of clot formation, and an integral component of thrombocyte aggregation. It has no alternatives, so it can be considered *primus inter pares* among the elements of the hemostatic system. Administering fibrinogen during bleeding is the first real substitutional step on the therapeutic pyramid (Gorlinger 2006; Gorlinger et al. 2011; Gorlinger et al. 2012; Gorlinger and Saner 2015; Gorlinger et al. 2013; Kozek-Langenecker et al. 2011). Depending on blood volume loss, fresh frozen plasma (FFP) may also be administered with fibrinogen concentrate (Rossaint et al. 2016; Babik et al. 2013; Fazakas et al. 2011).

Regarding factor supplementation, prothrombin complex concentrate contains four procoagulant factors (II−, VII−, IX−, and X) with anticoagulant elements (PC, PS, AT, and some heparin) in a balanced formula (4PCC). The main indication for 4PCC is to suspend coumarin effect or to replace vitamin-K-dependent factors in patients with liver disease who need an emergency surgical intervention (Rossaint et al. 2016; Babik et al. 2013). However, it can also be used for factor replacement during massive bleeding (Rossaint et al. 2016; Babik et al. 2013; Gorlinger et al. 2011). FFP may also be used for factor supplementation, with well-known disadvantages (Kozek-Langenecker 2016; Schochl et al. 2015). If the volume effect of FFP in massive bleeding is exploited or PCC-s are not available, solvent/detergent-treated plasma (S/D-FFP) may be advantageous. S/D-FFP has lower PS levels; therefore, its procoagulant characteristic is comparable to that of the plasma of pregnant women.

The decision to transfuse platelets should not be based solely on the absolute number of thrombocytes. If the patient is bleeding and the number of thrombocytes is < 100.000/μl, platelet transfusion is indicated (Rossaint et al. 2016; Babik et al. 2013). High fibrinogen level may compensate for the effects of thrombocytopenia (Babik et al. 2013). This may have importance when thrombocyte transfusion is difficult from a hematologic or logistic point of view.

If the patient is still bleeding after surgical, anesthesiological, and radiological efforts are exhausted, the next step might be off-label administration of recombinant activated factor VII (rFVIIa) (Rossaint et al. 2016). rFVIIa compels the patient’s own or already supplemented hemostatic system to operate via extrinsic activation. Therefore, all clinical and physiological hemostatic requirements should be secured before administering rFVIIa. Attention must be paid to older patients at higher risk for arterial occlusion complications. However, rFVIIa might be the only effective agent during critical perioperative bleeding. Under these desperate circumstances, it can be organ- or lifesaving. Because the decision must be made when massive transfusion is imminent, but before criteria for massive transfusion are met, it requires a highly trained perioperative team (Babik et al. 2013).

The replacement of factor XIII is at the top of the triangle. Since the onset time of FXIII is out of the focus and ranges of POC and traditional laboratory tests, FXIII deficit can only be deduced perioperatively (Dorgalaleh and Rashidpanah 2016).

## Diagnostic and therapeutic steps in managing major perioperative bleeding: complex algorithms of more recent guidelines

Evidence-based guidelines (Spahn et al. 2019; Rossaint et al. 2016; Kozek-Langenecker et al. 2017; Lier et al. 2016), a consensus statement (Munoz et al. 2019), reviews (Maegele et al. 2017; Schochl et al. 2012; Lier et al. 2013), and original articles (Monaco et al. 2020; Rigal et al. 2019; Schaden et al. 2012; Baksaas-Aasen et al. 2017) have recently become available regarding the management of massive bleeding following trauma (Spahn et al. 2019; Maegele et al. 2017; Rossaint et al. 2016; Schochl et al. 2012; Lier et al. 2013; Baksaas-Aasen et al. 2017) and cardiac (Monaco et al. 2020; Rigal et al. 2019; Scala et al. 2020), obstetrical (Lier et al. 2016; Munoz et al. 2019), and general surgery (Kozek-Langenecker et al. 2017). These publications integrate POC viscoelastic tests to individualize administration of quantified prothrombotic agents and contain more comprehensive recommendations. Their structure still recalls the fundamental points of gestational hemostasis and the triangle of hemostatic interventions detailed above (Fig. 1C).

Initially, if severe intraoperative bleeding is of anatomical origin, it must be treated appropriately (Spahn et al. 2019; Rossaint et al. 2016; Schochl et al. 2012; Kozek-Langenecker et al. 2017; Munoz et al. 2019; Lier et al. 2013; Monaco et al. 2020; Schaden et al. 2012; Baksaas-Aasen et al. 2017).

The importance of hemoglobin and Ca^++^ level, core temperature, and pH is underlined in the coagulation process (Spahn et al. 2019; Maegele et al. 2017; Rossaint et al. 2016; Schochl et al. 2012; Kozek-Langenecker et al. 2017; Lier et al. 2016; Munoz et al. 2019; Lier et al. 2013; Monaco et al. 2020; Rigal et al. 2019; Schaden et al. 2012).

Hyperfibrinolysis is a consequence of major tissue trauma. Obtaining direct lysis parameters via rotational thromboelastometry may not be quick enough to assess the severity of fibrinolysis (Maegele et al. 2017; Lier et al. 2013; Rigal et al. 2019; Schaden et al. 2012; Baksaas-Aasen et al. 2017). Therefore, early administration of antifibrinolytics is strongly recommended (Spahn et al. 2019; Maegele et al. 2017; Rossaint et al. 2016; Schochl et al. 2012; Kozek-Langenecker et al. 2017; Lier et al. 2016; Munoz et al. 2019; Lier et al. 2013; Monaco et al. 2020; Rigal et al. 2019; Schaden et al. 2012; Baksaas-Aasen et al. 2017; Scala et al. 2020).

Administering fibrinogen is an essential component of the goal-directed hemostasis resuscitation strategy (Spahn et al. 2019; Rossaint et al. 2016; Kozek-Langenecker et al. 2017; Munoz et al. 2019). The comparison of the clot amplitude measured by extrinsically triggered rotational thromboelastometry (EXTEM) and cytochalasin D added EXTEM test (FIBTEM) at 5 (Scala et al. 2020) or 10 min (Maegele et al. 2017; Monaco et al. 2020; Rigal et al. 2019; Schaden et al. 2012) or at maximum clot strength (Lier et al. 2013) can reveal the lack of fibrinogen. Similarly, the difference in maximal clot amplitude measured by rapid thromboelastography (r-TEG) and functional fibrinogen TEG (FF-TEG) can also indicate fibrinogen deficit (Rigal et al. 2019; Baksaas-Aasen et al. 2017). Low clot amplitude with FIBTEM at five minutes (Munoz et al. 2019) may also indicate depleted fibrinogen level. In the absence of viscoelastic assays, traditional laboratory tests are suggested (Spahn et al. 2019; Maegele et al. 2017; Rossaint et al. 2016; Kozek-Langenecker et al. 2017; Munoz et al. 2019; Rigal et al. 2019).

The viscoelastic measurements can also reflect the efficiency of thrombocytes via a decreased difference between EXTEM and INTEM (Maegele et al. 2017; Schochl et al. 2012; Monaco et al. 2020; Rigal et al. 2019; Schaden et al. 2012; Baksaas-Aasen et al. 2017; Scala et al. 2020) or between r-TEG and FF-TEG (Rigal et al. 2019; Baksaas-Aasen et al. 2017). The number and functionality of platelets can also be assessed by a traditional platelet count (Spahn et al. 2019; Maegele et al. 2017; Rossaint et al. 2016; Kozek-Langenecker et al. 2017; Munoz et al. 2019; Rigal et al. 2019) or aggregometry tests (Rossaint et al. 2016; Kozek-Langenecker et al. 2017; Lier et al. 2013).

A defect in thrombin generation can be attributed to a deficiency of factors often reflected in the prolongation of EXTEM clotting time (CT) (Maegele et al. 2017; Schochl et al. 2012; Lier et al. 2013; Monaco et al. 2020; Rigal et al. 2019; Schaden et al. 2012; Baksaas-Aasen et al. 2017; Scala et al. 2020).

rFVIIa is recommended only as an off-label treatment for severe bleeding that persists despite all surgical and medical hemostasis attempts (Maegele et al. 2017; Schochl et al. 2012; Lier et al. 2013; Rigal et al. 2019; Scala et al. 2020).

After major surgery or trauma, decreased levels of factor XIII might be responsible for bleeding despite normal viscoelastic parameters. Thus, administration of FXIII may increase clot stability (Kozek-Langenecker et al. 2017; Lier et al. 2013).

### Global hemostatic changes in pregnancy

Global gestational hemostasis rebalances the dynamic equilibrium of hemostatic processes in time and space (Fig. 1A). The hemostatic shift with strong uterine contractions immediately after delivery prevents peripartum bleeding (Bremme 2003; Cerneca et al. 1997; Faught et al. 1995; Greer 1994; Isermann et al. 2003; Lefkowitz et al. 1996; Stirling et al. 1984; Kruithof et al. 1987; Thornton and Douglas 2010; Hellgren 2003; Uchikova and Ledjev 2005; Valera et al. 2010). At term, placental blood flow is comparable to the femoral arterial flow (650–700 ml/min) (Astedt et al. 1986). Therefore, potential bleeding resulting from placental abruption could theoretically cause exsanguination similar to that of a two-sided femoral artery injury. Consequently, the natural, programmed complex hemostatic protection dramatically decreases the mortality and morbidity at delivery.

The aggregation–antiaggregation balance is not affected in pregnancy per se. The size, functionality, and lifetime of thrombocytes do not change, but their number may decrease during the third trimester (Valera et al. 2010)_._ This can partly be attributed to dilution (Carlin and Alfirevic 2008). However, the thrombocytes’ potential for adhesion and aggregation is facilitated by doubled levels of von-Willebrandt factor (vWF) and fibrinogen at term. Consequently, the aggregation-antiaggregation processes are balanced, but with an increased aggregational potential.

The coagulation–anticoagulation balance is shifted progressively toward a prothrombotic potential. The level of factor VII (FVII) may be up to three times higher (Bremme 2003; Stirling et al. 1984; Thornton and Douglas 2010; Uchikova and Ledjev 2005). Plasma fibrinogen-concentration increases by 50%; consequently, the amount is doubled due to the higher plasma volume (Bremme 2003; Stirling et al. 1984), and the levels of factors II−, V−, VIII−, and X also increase slightly (Bremme 2003). Only the levels of factors XI- and XIII decrease (Coopland et al. 1969), and elevated level of FXIII would impede emptying the uterus postpartum. There is no change in the anticoagulant protein C (PC) level (Faught et al. 1995). However, the protein S (PS) level decreases (Lefkowitz et al. 1996), while antithrombin (AT) remains unchanged (Bremme 2003); only the soluble thrombomodulin level increases slightly (Hellgren 2003). Thus, the coagulation–anticoagulation processes are still balanced, but a strong procoagulant reserve related to the extrinsic and common pathway is accumulated.

Expression of the fibrinolytic tissue plasminogen activator (t-PA) level can be decreased or slightly increased during pregnancy (Hellgren 2003; van Wersch and Ubachs 1991). The plasma level of plasminogen increases (Hellgren 2003), highlighting the physiologic mechanism of establishing substrate reserves before a timed enzyme mechanism embedded in a stress (i.e., delivery) process. Plasminogen activator inhibitor-1 (PAI-1) triples at the end of pregnancy (Bremme 2003), and the synthesis of pregnancy-specific plasminogen activator inhibitor-2 (PAI-2) is also significant (Kruithof et al. 1987; Hellgren 2003). Furthermore, fibrinolysis is inhibited globally by slightly elevated α_2_-antiplasmin and locally by thrombin activated fibrinolysis inhibitor (TAFI) (Hellgren 2003). Thus, the fibrinolytic–antifibrinolytic balance is shifted to antifibrinolytic dominance during pregnancy until the end of delivery. However, high plasminogen level forms a significant fibrinolytic potential with local and global importance in resolving uterine clot and preventing thrombosis after delivery, respectively.

Thus, the global gestational hemostasis is well balanced with a huge prothrombotic reserve to prevent maternal bleeding at delivery. This strong prothrombotic potential is controlled by the high levels of the first cascade-elements reacting to the trigger of the aggregation, coagulation, and fibrinolytic processes (von-Willebrandt factor, FVII, t-PA, PAI-1/2) and the final volume-substrates of these cascades and (fibrinogen, plasminogen).

## Comparison of the early goal-directed factor-based therapeutic hemostasis resuscitation approaches and gestational hemostasis

The algorithms refer to the diagnostic and therapeutic steps of hemostatic resuscitation during massive bleeding, whereas global gestational hemostasis prepares the mother to prevent massive bleeding during delivery. The former is a set of diagnostic and therapeutic interventions, the latter is a natural process; the former treats, the latter prevents, but their physiologic rationale is similar, to facilitate balanced building prothrombotic potential. Thus, *most of the steps of the therapeutic recommendations can be matched with the “successful natural approach.”* These analogies between the therapeutic and the gestational hemostatic approaches (Fig. 1, arrows) highlight several important clinical aspects.

The algorithms emphasize the role of adequate surgical hemostasis as first line therapy. This basic recommendation is functionally analogous to the contraction of the uterine muscles after delivery, which leads to mechanical closure of the open, large spiral arteries.

The therapeutic algorithms aim to achieve an optimal homeostatic environment. The adaptive changes during pregnancy point an identical homeostatic goal.

The algorithms suggest attenuating fibrinolysis by administering TXA. Gestational hemostasis is also characterized by low level fibrinolysis mediated by the depressed expression of t-PA and elevated levels of PAI-1 and PAI-2. The level of α_2_-antiplasmin and TAFI are also increased.

Supplementation of fibrinogen is an emphatic substitutional step of the diagnostic and therapeutic algorithms. Similarly, the relative and absolute elevation of fibrinogen is a hallmark of pregnancy.

If bleeding persists, algorithms recommend administration of factors to correct missing coagulation factors. During pregnancy, levels of these factors are increased as well.

Diagnostic and therapeutic proposals may suggest thrombocyte transfusion. During massive bleeding, platelets can be mobilized from the spleen, and endothelium expresses vWF. The goal of these steps, together with the patient’s own compensatory reactions, is to attain the aggregational status seen in pregnancy (i.e., elevated vWF and close to normal platelet level).

If bleeding persists despite the best clinical practice, off-label rFVIIa administration can be considered. It is noteworthy that the increase in FVII concentration is the highest among the plasma coagulation factors in pregnancy at term, which allows for a fast and efficient response to the high amount of TF following placental abruption, resulting in rapid local clot formation.

Administration of FXIII is rarely recommended by the algorithms. Similarly, at term, FXIII decreases by ~ 50%, facilitating postpartum clot elimination. High amount of FXIII would impede clot dissolving leading to complications and pain.

The prothrombotic standby state of gestational hemostasis at term underlines the fact that a multicomponent biological system can only be effective if all the elements are available simultaneously. This “philosophy of the physiology” has a clear message to the clinician. Consequently, all components judged necessary by traditional or POC tests should be administered within ~ 30 min, not by portions, which would preserve the shortages in a dangerous and costly manner.

Periprocedural bleeding cannot be predicted; thus, the mechanism of gestational accumulation of prothrombotic reserves is not adaptable for the perioperative care of massive bleeding per se. Nevertheless, the time of severe hemorrhage can be often anticipated, such as in complex cardiac, vascular, or hepatic surgery. In these cases, supplementing the estimated losses of prothrombotic components may start immediately after correcting surgical bleeds. This management enables *not only treat* but to *prevent* massive bleeding and can be considered as secondary prevention of further severe periprocedural bleeding. This is an important difference in morbidity and mortality for patients, and it is of great value for health care providers. Furthermore, the analogy for prothrombotic reserves is still valid in term of in vitro reserves. Pharmacological hemostatic products must be stored and be readily available at bedside.

The marked increase of FVII during pregnancy suggests its substantial role in restricting massive bleeding at delivery. Considering natural gestational hemostasis as a model, administration of rFVIIa needs discussion in situations of critical persistent life-threatening perioperative blood loss. In such desperate cases, the risk of arterial occlusion with rFVIIa use is outweighed by the risk of death (Rossaint et al. 2016; Babik et al. 2013; Vincent et al. 2006). The off-label and *ultimum refugium* application of rFVIIa emerges particularly during life-threatening bleeding in young patients (Vincent et al. 2006). The physiology and pharmacology of FVII and rFVIIa might help clarify this dilemma. The FVII plasma concentration is normally 500 ng/ml, and only 1% (5 ng/ml) is active (Moor et al. 1995). Pregnancy increases the inactive form substantially; thus, a huge quantity of FVII is ready to bind to the tissue factor expressed locally in large amounts after placental abruption, and the TF-FVIIa complex is subsequently launches extrinsic coagulation pathway. As the circulating plasma volume is 3–4 L, administering 2 mg rFVIIa *iv.* elevates the plasma concentration of the active form 100 times higher than normal. Therefore, the risk of any arterial occlusion seems to be plausible, but physiology provides the answer. The activation of FX in the extrinsic pathway by the TF-FVIIa binary complex is 1000 times more effective than by FVIIa alone (Banner et al. 1996; Roberts 2006). Thus, FVIIa alone is not sufficient to initiate coagulation; combination with TF is needed. Therefore, rFVIIa acts locally at the site of TF expression. Nevertheless, in elderly atherosclerotic patients, damaged endothelium may release TF elsewhere, thereby risking arterial occlusion. However, too many young women giving birth as well as young trauma patients have already been saved by *off-label* use of rFVIIa to refuse its application in life-threatening situations ab ovo*.* More studies are needed to establish the role of rFVIIa in perioperative settings (Vincent et al. 2006), studying gestational hemostasis.

The natural protective mechanisms against bleeding work on different levels. Small injuries activate only the primary hemostasis, while moderate wounds activate more complex mechanisms (primary, secondary, tertiary hemostasis). As a third level, a high capacity, rebalanced hemostatic system with huge prothrombotic reserves is developed by pregnant women. These categories of the protecting power should be considered perioperatively as well. Massive bleeding requires *complex and complete* hemostatic resuscitation. Incomplete supplementation will result in a low concentration of prothrombotic components, and the missing links inhibit the hemostatic system to function as a whole. Patients with quantitative and qualitative defects might be treated but not cured.

Gestational hemostasis can physiologically break the massive bleeding-massive transfusion coupling. The application of the principles of this “natural approach” may help prevent massive bleeding. It highlights that management of massive bleeding might be possible without a transfusion of huge quantities of allogenic blood products *if enough procoagulant components are given with the right timing.* Therefore, European guidelines propose early goal-directed individualized factor-based hemostasis resuscitation, leaving a high RBC:FFP ratio to be applied in a context-sensitive way, i.e., depending on the volume status of the severely bleeding patient (Rossaint et al. 2016; Schochl et al. 2012; Babik et al. 2013; Kozek-Langenecker et al. 2017; Munoz et al. 2019).

Studying gestational hemostasis facilitates natural based, *less invasive mechanisms* of clinical management for perioperative massive bleeding applying less homologous blood, and thus may *guide future practice.*

## Conclusions

There are remarkable analogies between the early goal-directed clinical management of severe bleeding and the mechanisms of global gestational hemostasis. Therefore, hemostatic changes in pregnancy may serve as a natural model for periprocedural hemostasis resuscitation. With the application of the “philosophy of physiology,” the massive tissue injury-massive bleeding-massive transfusion coupling can be broken, making hemostasis resuscitation a natural-based, less invasive procedure saving more blood and lives.

## Data Availability

Not applicable.

## Abbreviations

ADP: Adenosine diphosphate
AT: Antithrombin
CT: Clotting time
EXTEM: Extrinsically activated rotational thromboelastometry test
FFP: Fresh frozen plasma
FF-TEG: Functional fibrinogen TEG
FIBTEM: Extrinsically activated test with platelet inhibitor cytochalasin D
FII: Factor II
FV: Factor V
FVII: Factor VII
FVIII: Factor VIII
FX: Factor X
FXI: Factor XI
FXIII: Factor XIII
GP-Ib: Glycoprotein-Ib
GP-IIb-IIIa: Glycoprotein-IIb-IIIa
INTEM: Intrinsically activated rotational thromboelastometry test
PAI-1: Plasminogen activator inhibitor-1
PAI-2: Plasminogen activator inhibitor-2
PC: Protein C
PCC: Prothrombin complex concentrate
POC: Point-of-care
PS: Protein S
RBC: Red blood cell
*rFVIIa*: Recombinant activated factor VII
ROTEM: Rotational thromboelastometry
r-TEG: Rapid TEG
S/D-FFP: Solvent/detergent-treated plasma
TAFI: Thrombin activated fibrinolysis inhibitor
TAT: Thrombin-anti-thrombin complexes
TEG: Thromboelastography
TF: Tissue factor
t-PA: Tissue plasminogen activator
TXA: Tranexamic acid
vWF: Von-Willebrandt factor
4PCC: Prothrombin complex concentrate containing four coagulations factors
